# A Review on Ethnopharmacological Applications, Pharmacological Activities, and Bioactive Compounds of* Mangifera indica* (Mango)

**DOI:** 10.1155/2017/6949835

**Published:** 2017-12-31

**Authors:** Meran Keshawa Ediriweera, Kamani Hemamala Tennekoon, Sameera Ranganath Samarakoon

**Affiliations:** Institute of Biochemistry, Molecular Biology and Biotechnology, University of Colombo, 90 Cumaratunga Munidasa Mawatha, Colombo 03, Sri Lanka

## Abstract

*Mangifera indica* (family Anacardiaceae), commonly known as mango, is a pharmacologically, ethnomedically, and phytochemically diverse plant. Various parts of* M. indica* tree have been used in traditional medicine for the treatment of different ailments, and a number of bioactive phytochemical constituents of* M. indica* have been reported, namely, polyphenols, terpenes, sterols, carotenoids, vitamins, and amino acids, and so forth. Several studies have proven the pharmacological potential of different parts of mango trees such as leaves, bark, fruit peel and flesh, roots, and flowers as anticancer, anti-inflammatory, antidiabetic, antioxidant, antibacterial, antifungal, anthelmintic, gastroprotective, hepatoprotective, immunomodulatory, antiplasmodial, and antihyperlipemic. In the present review, a comprehensive study on ethnopharmacological applications, pharmacological activities, and bioactive compounds of* M. indica* has been described.

## 1. Introduction

*M. indica* L. is considered as one of the main tropical fruits in the world believed to be originated from Asia [1]. It has been reported that China, India, Brazil, Nigeria, Pakistan, Mexico, Thailand, and Philippine are well-known for mango cultivation with India being the highest mango cultivating country [2]. World production of mango is approximately 42 million tons per year which is second only to banana production. There are about 1000 mango varieties grown all over the world [2–4]. Mango is known by various names around the world, for example, Manja in Arabic, Mannko in Greek, Am or Ambi in Hindi, Amba in Sinhala, Mangue in French, Mango in Finnish, Mango in Dutch, Mangue in German, Mángguǒin in Chinese, and Mampalam in Tamil [5]. Both ripe or unripe mango fruits are in human use as pickles, juice, oils, nectar, powder, sauce, cereal flakes, and jam [6]. Mango fruit peel and flesh are reported to be a rich source of fiber, vitamin C and A, essential amino acids, and polyphenols [7]. Mango seed has also been reported as a rich source of polyphenols [8]. Despites the common use of mango fruit as a food item, various parts of mango trees have also been used for medical purposes since ancient times, mostly in Southeast Asian and African countries [9]. Much evidence is found in literature on pharmacological and ethnomedicinal uses of* M. indica*; however, there is no complete review on phytochemicals, biological effects of phytochemicals, and pharmacological and ethnomedicinal properties of* M. indica*. Therefore, we present this review as an up-to-date and comprehensive evaluation which mainly includes phytochemicals, some reported bioactivities of phytochemicals, and pharmacological and ethnomedicinal properties of* M. indica*.

## 2. Taxonomy and Botanical Description of* M. indica*

**Figure 1:**
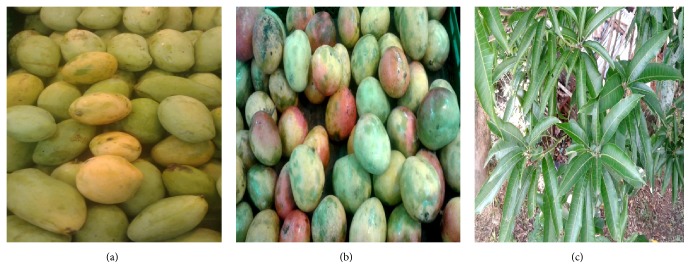
*M. indica* fruits ((a) and (b)) and leaves (c).

The genus* Mangifera* belongs to the family Anacardiaceae. Genus* Mangifera* approximately contains 69 different species with* M. indica* being the most common species in the same genus [10, 11].* M. indica* plant is an evergreen broad canopy tree which grows to a height of 8–40 m [12].* M. indica* bark is a thick brown-gray colour and is superficially cracked [13]. Leaves are 15–45 cm in length with variable sizes [13]. Leaf petiole has a variable length from 1–10 cm [13].* M. indica* leaves (Figure 1) possess different shapes (lanceolate, ovate-lanceolate, linear-oblong, roundish-oblong, oval, and oblong) [13]. Green, red, and yellow leaves are seen in some mango varieties and upper leaf surfaces are normally shiny [13, 14]. In case of* M. indica* flowers, male and hermaphrodite flowers are produced in the same panicle; its size can vary from 6–8 mm in diameter. There are about 4000–5000 small flowers in panicles with red/purple spots on petals [13, 14]. Even though a large number of flowers present in panicles, very few will be developed as fruits. Flowering season is mainly from January to April and most of the flowers are subsessile and have a sweet smell.* M. indica* fruit (Figure 1) is drupe with different sizes, shapes, and colours. Fruit peel is green, yellow, red, or orange. Seeds are ovoid- or oblong-shaped covered with a hard endocarp having a woody fiber covering [15].

## 3. Ethnomedicinal Use

Various parts of* M. indica* (bark, leaves, roots, fruits, and flowers) have been used in traditional medicine for the treatment of various diseases and conditions. Ethnomedicinal uses of various parts of* M. indica* in different countries in the world have been summarized in Table 1.

## 4. Chemistry

### 4.1. Phytochemicals in* M. indica*

**Figure 2:**
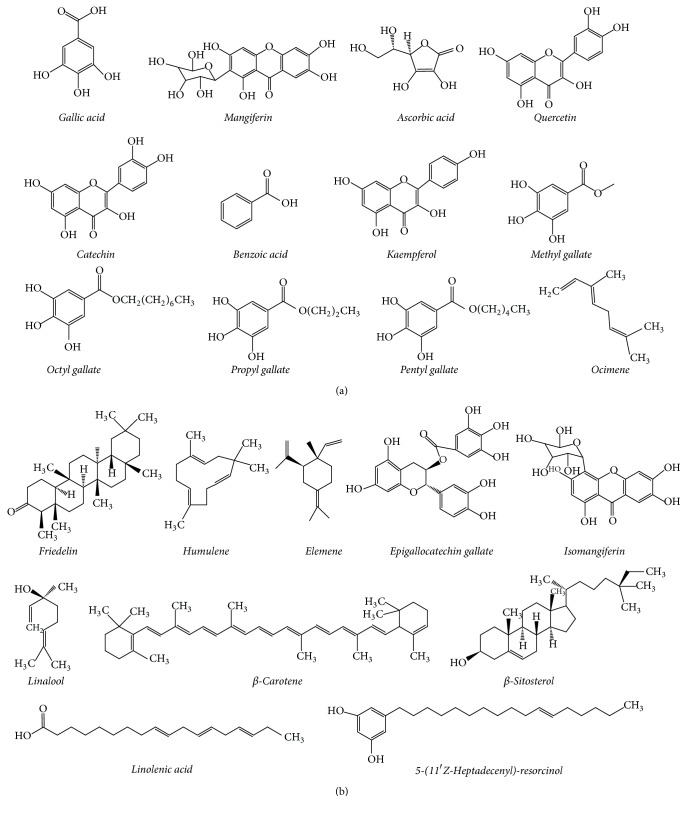
(a and b) Structures of some common compounds present in* M. indica*.

A large variety of chemical compounds have been reported in* M. indica* [30]. Among these, polyphenols (flavonoids, xanthones, and phenolic acids) are the most abundant compound types in* M. indica* [31]. Mangiferin, gallic acid, catechins, quercetin, kaempferol, protocatechuic acid, ellagic acids, propyl and methyl gallate, rhamnetin, and anthocyanins are the major polyphenolic compounds found in* M. indica* [32]. Mangiferin is a well-known polyphenolic compound which has been extensively studied for its numerous biological properties [33]. The quantities of different polyphenols in mango depend on the part and variety of mango [34]. Antioxidant properties have been shown to be the main biological property of almost all the* M. indica* polyphenols [35]. Ascorbic acid and dehydroascorbic acid (oxidized form of ascorbic acid) are two other common polyphenols found in* M. indica* [36]. The amount of polyphenols is high in many parts of* M. indica*. Thus, a pure compound alone has been proven to be less effective than crude drugs, implying that the synergism of many* M. indica* polyphenols is essential for optimum biological activities [37, 38]. Carotenoids are another class of natural compounds found in plants. They are considered as natural organic pigments [39]. The bright yellow colour of* M. indica* fruit peel and flesh is due to the presence of carotenoids [39]. Biologically they are very good free radical scavengers [40]. It has been reported that carotenoids in* M. indica* are biosynthesized in the fruit and carotenoid concentration rises upon ripening [41]. *β*-carotene, luteoxanthin, violaxanthin, neoxanthin, zeaxanthin, and cryptoxanthin are the main carotenoids found in* M. indica* fruit flesh and peel [42]. Among these, *β*-carotene is the most abundant [42]. Terpenoids are a class of lipids, similar to terpenes, commonly found in the plant kingdom [43].* M. indica* is reported to contain several terpenoids, including careen, ocimene, terpinolene, myrcene, or limonene [44]. These terpenoids are volatile and responsible for aroma in* M. indica* [45]. Lupeol and lupeollinoleate are two other common triterpenoids found in mango [46]. Gallotannins (hydrolyzable tannins) are another class of chemical compounds found in* M. indica* bark, leaves, kernel, and fruit pulp [47]. The presence of tocopherols in* M. indica* has also been reported. Alpha-tocopherol, beta-tocopherol, and gamma-tocopherol are commonly found tocopherols in* M. indica* fruit peel and flesh [48]. Resorcinolic lipids (phenolic lipids) are another class of natural compounds found in* M. indica* [49]. The isolation of a wide range of resorcinolic lipids with different biological properties has been reported from* M. indica* fruit peels, flesh, and bark [49]. The isolation of a novel resorcinolic lipid from the bark of* Mangifera zeylanica* (endemic Sri Lankan mango) with anticancer effects has been presented in a study carried out by us [50]. It was thought that halogenated compounds are limited only to marine plants and microorganisms. However, occurrence of halogenated compounds in the bark of* M. indica* has been reported in a study conducted in India [51]. A recent study carried out by us also reported the isolation of two novel halogenated compounds (chloromangiferamide and bromomangiferic acid) from the bark of* M. zeylanica* [52]. Structures of some common compounds present in* M. indica* are shown in Figures 2(a) and 2(b). Quercetin and mangiferin are most commonly found in* M. indica*. As these two compounds containing food items (including mango fruits) are very common in human diet, studies on their safety and toxicity have been well-documented [17, 53–55]. Moreover, kaempferol, another well-known mango compound, has also been subjected to various safety and toxicity studies in order to validate its uses in human diet [56–58]. In most of the studies it has been mentioned that quercetin mangiferin and kaempferol are less toxic in studied animal models.

### 4.2. Reported Phytochemicals in Different Parts of* M. indica*

#### 4.2.1. Leaves

Amino acids include alanine, glycine, valine, tyrosine, leucine, and *γ*-aminobutyric acid. Polyphenols and phenolic acids include protocatechuic acid, gallic acid, hyperin, catechin, quercetin, mangiferin, kainic acid, ethyl digallate, ellagic acid, and shikimic acid. Alcohols include methylic, ethyl, and isobutyl alcohols. Terpenes include *α*-pinene, *β*-pinene, *δ*-elemene, taraxerol, *β*-elemene, *α*–cubebene, camphene, *γ*–cadinene, lupeol, friedelin, linalool, *β*-bulnesene, *α*-guaiene, humulene, *α*-farnesene, myrcene, car-3-ene, limonene, *β*-ocimene, *γ*–terpinene, and *α*-terpinolene. Phenylpropenes include estragole, methyleugenol and elemicin. Sterols include *α*, *β*, and *γ*-sitosterol [59–64].

#### 4.2.2. Fruit Peel and Flesh

Triterpenes and triterpenoids include cycloartenol, *α*-amyrin, *β*-amyrin, ocotillol, 3b-hydroxycycloart-24-en-26-al, 24-methylene-cycloartan-3b,26-diol, dammarenediol II, and psi-taraxastane-3b. Polyphenols and phenolic acids include ascorbic acid, quercetin, mangiferin, quercetin 3-ara, quercetin 3-rha, isomangiferin gallate, mangiferin gallate, methyl mangiferonate, methyl mangiferolate, tetra-O-galloylglucose, hexa-O-galloylglucose, methyl isomangiferolate, caffeic acid, ferulic acid, gallic acid, cinnamic acid, vanillin, rhamnetin-3-O-galactoside, kaempferol, and kaempferol-hexose. Resorcinolic lipids include 5-(11′Z-heptadecenyl)-resorcinol and 5-(8′Z,11′Z-heptadecadienyl)-resorcinol. Carotenoids include *β*-carotene, cis-violaxanthin, neochrome, cis-neoxanthin, luteoxanthin, zeaxanthin, and 9- or 9′-cis-lutein. Long-chain fatty acids include oleic acid, linoleic acid, linolenic acid, and n-pentacosanol [49, 59–64].

#### 4.2.3. Root

Triterpenes and triterpenoids include friedelin, friedelan-3b-ol, *α*-amyrin, *β*-amyrin, and cycloartenol. Sterols include *β*-sitostero and 3-methoxy-2-(4′-methyl benzoyl)-chromone [59–64].

#### 4.2.4. Bark

Polyphenols and phenolic acids include protocatechuic acid, catechin, mangiferin, benzoic acid, kainic acid, gallic acid, shikimic acid, and kaempferol. Triterpenes and triterpenoids include cycloart-24-en-3b,26-diol, 3-ketodammar-24(E)-en-20S,26-diol, friedelin, mangocoumarin, manglupenone, manghopanal, cycloartan-3*β*-30-diol cycloartan-3b,24,27-triol, mangoleanone, mangiferolic acid ethyl ester, mangiferolate A and mangiferolate B, and 29-hydroxymangiferonic acid. Halogenated amide includes 3-chloro-N-(2-phenylethyl) propenamide. Long-chain hydrocarbons include N-triacontane, N-tetracosane, and 9,12-tetradecadiene-1-ol-acetate. Terpenoid saponins include indicoside A and B. Amino acids include alanine, glycine, and *γ*-aminobutyric acid [51, 59–65].

#### 4.2.5. Seed and Kernel

Long-chain hydrocarbons and fatty acids include stearic acid, eicosanoic acid, linoleic, linolenic, oleic acid, arachidonic acid, and palmitic acid. Sterols include stigmasterol, sitosterol, and campesterol. Triterpenes and triterpenoids include *α*-pinene, *β*-pinene, myrcene, and limonene. Polyphenols and phenolic acids include ascorbic acid, mangiferin, quercetin, and gallic acid [59–67].

#### 4.2.6. Flowers

Amino acids include threonine, valine, alanine, and tryptophan. Polyphenols and phenolic acids include gallic acid, mangiferin, quercetin, and ellagic acid. Triterpenes and triterpenoids include *β*-pinene, nerol, limonene, *α*-phellandrene, and *α*-pinene [59–68].

## 5. Pharmacological Properties of* M. indica*

A number of* in vitro* and* in vivo* studies have been carried out to reveal various pharmacological potentials of* M. indica*. Different parts of* M. indica* trees have been demonstrated to exert anticancer, anti-inflammatory, antidiabetic, antioxidant, antibacterial, antifungal, anthelmintic, gastroprotective, hepatoprotective, immunomodulatory, antiplasmodial and antihyperlipemic effects [69]. Many of these pharmacological studies on different parts (as organic extracts or decoctions) of* M. indica* trees have been carried out to validate the ethnomedical uses of the plant in traditional medicine in the treatment of several diseases and conditions. A large number of pharmacological studies on* M. indica* have been conducted mainly in India and Bangladesh. A considerable number of pharmacological studies have also been reported from countries like Brazil, Nigeria, and Iran. Some experimentally proven pharmacological properties of different parts of* M. indica* trees have been described in detail in the following section.

### 5.1. Antioxidant Properties of* M. indica*

Antioxidants are substances which inhibit/delay oxidative damage by trapping free radicals to a target molecule [70]. Several classes of natural compounds including polyphenols, phenolic acids, and flavonoids are reported as good free radical scavengers [71]. It has been reported that reactive oxygen species (ROS) and some other oxidants cause various disorders and diseases to human [71]. Humans possess antioxidative mechanisms which fight against reactive oxygen species (ROS) and some other oxidants by deactivating free radicals before they attack targets in human body [72]. Naturally occurring antioxidants have gained much attention recently as they possess a remarkable ability to fight against free radicals and reactive oxygen species [72]. As almost all the parts of the mango tree are reported to possess polyphenols, which are well-known antioxidants, most of the pharmacological studies have proven that antioxidant properties with extract(s) of various parts of the* M. indica* tree are related to polyphenolic content. The following section will summarize some selected studies which illustrate antioxidant effects of different parts of* M. indica*. A recent study carried out by Thambi et al. 2016 [73] evaluated antioxidant effects of mango peel powder and proved that the acetone extract of the* M. indica* peel exerts strong radical scavenging effects. A research carried out by Abbasi et al. 2017 [74] with nine mango varieties (Royal mango, Thai mango, Egg mango, Luzon, Narcissus, Big Tainong, Keitt, Australian mango, and Small Tainong) found in China found that the peel of Small Tainong (Xiao Tainong) variety exerts the highest antioxidant potential among the tested varieties. Another study conducted by Sultana et al. 2012 [75] measured the antioxidant potential of water-methanol extracts of the peels of two mango varieties (Langra and Chaunsa) grown in Pakistan. Among these two peel extracts, water-methanol extract of Chaunsa exhibited strong antioxidant effects than Langra. Kim et al. 2010 [76] who studied antioxidant effects of ethanolic extracts of mango peel and flesh showed potent antioxidant effects of mango peel extracts compared to flesh extracts. Effect of temperature on the antioxidant activity of mango peel extracts (a variety found in Spain) was studied by Dorta et al. 2012 [77]. Methanol, ethanol, acetone, water, methanol-water, acetone-water, and ethanol-water extracts were subjected to radical scavenging activity and methanol-water, acetone-water and ethanol-water extracts were found to possess high antioxidant capacity when the temperature is increased from 50−70°C. A number of studies around the world have evaluated the antioxidant effects of mango fruit flesh. A study conducted with methanol extracts of fruit flesh of two mango varieties (Americana and Jose) grown in France by Septembre-Malaterre et al. 2016 [78] has shown that methanol extracts of both these varieties exert antioxidant effects. A comparative study has been carried out with fruit flesh of five mango varieties (Langara, Fazli, Amrupali, Himsagor, and Ashwina) grown in Bangaladesh [79]. Methanolic extracts of all the tested mango varieties exerted considerable antioxidant effects and the fruit flesh extract of Langra exerted the highest effect. Another comparative study carried out with methanol/dichloromethane and aqueous extracts of fruit flesh of four Egyptian mango varieties (Zebdia, Sukkari, Taimor, and Hindi) showed that methanol/dichloromethane extracts of all the varieties have prominent antioxidant effects than the aqueous extracts [80]. Antioxidant potential of ethanolic extract of the seed of an* M. indica* variety grown in Malaysia has been reported by Norshazila et al. 2010 [81]. Pitchaon, 2011 [82], has demonstrated antioxidant capacity of seed kernel obtained from a mango variety (Chok-Anan) grown in Thailand. Two kernel extracts have been prepared by acid hydrolysis and shaking in ethanol. The results of this study showed that the extract prepared by acid hydrolysis has a high antioxidant potential than the extract prepared by shaking in ethanol. Sultana et al. 2012 [75] have presented antioxidant potential of water-methanol extracts of the bark of two* M. indica* varieties (Langra and Chaunsa) grown in Pakistan. The bark of the variety Chaunsa exerted a higher antioxidant potential than Langra. Antioxidant effects of methanolic extract of* M. indica* leaves have been reported by Mohan et al. 2013 [83]. They showed that ethyl acetate and butanol fractions obtained after solvent partition of the crude methanol extract have antioxidant effects.

### 5.2. Anti-Inflammatory Effects of* M. indica*

Several naturally found polyphenols are reported to possess anti-inflammatory effects via inhibition of nuclear factor kappa-B (NF-*κ*B) [84]. However, anti-inflammatory activities of these compounds depend on their chemical structures and their cellular targets [84]. Production of a large amount of proinflammatory cytokines (IL-1, 2 and 6 and TNF) increase the expression of enzymes such as COX-2 and iNOS which are associated with anti-inflammations [85]. Nuclear factor kappa-B (NF-*κ*B), a transcriptional factor, is reported to control expression of proinflammatory cytokines [86]. Ulcerative colitis and inflammatory bowel disease are considered as main diseases that occur due to chronic inflammation [87]. Several studies have shown that mango extracts can exert anti-inflammatory effects in experimental models of ulcerative colitis. In a recent study, treatment with a mango beverage prepared from fruit (Mexican variety) which consists of polyphenols and vitamins has caused attenuation of colitis symptoms by expressing the PI3K/AKT/mTOR pathway [88]. Another study conducted by the same authors showed that the same mango polyphenols-rich beverage can inhibit the IGF-1R/AKT/mTOR pathway in ulcerative colitis [89]. In another study, aqueous extract of stem-bark extract from* M. indica* rich in polyphenols and flavonoids was found to attenuate colitis symptoms in a model of colitis [90]. Attenuation of symptoms was accompanied by a reduction in COX-2, TNFR-2, TNF-*α*, and iNOS levels in colonic tissue. Gout is considered as one of the most common causes of inflammatory arthritis. Deposition of monosodium urate crystals on local tissue and joints is the major clinical manifestation of gout [91]. Antigouty arthritis effects of ethanol extract of* M. indica* leaves have been studied by Jiang et al. 2012 [91]. Oral administration of ethanol extract of* M. indica* leaves has caused reduction in IL-1*β* and TNF-*α* mRNA levels and ankle swelling in a rat with gouty arthritis induced by monosodium urate [91].

Vimang is an aqueous extract of* M. indica* (stem-bark) used in Cuba as a natural supplement [92]. A number of* in vivo* and* in vitro* studies have been conducted with Vimang to demonstrate its antioxidant effects. A study carried out by Garrido et al., 2004 [92], has shown that administration of Vimang can reduce arachidonic acid (AA) and phorbolmyristate acetate-induced ear edema in mice. Reduction in myeloperoxidase (MPO) activity was observed in phorbolmyristate acetate-induced mice. Inhibition of tumor necrosis factor alpha (TNF) serum levels was also observed in both models of inflammation after administration of Vimang.* In vitro* evaluations carried out with Vimang have shown that it can inhibit PGE2 (prostaglandin E2) or LTB4 (Leukotriene B4) in macrophage cells (RAW264.7) induced with proinflammatory stimuli. Another study conducted by Garrido et al., 2001 [93], has also shown possible anti-inflammatory effects of Vimang. Carrageenan and formalin-induced oedema in mice were used to study anti-inflammatory effects in this study. Results of this study have shown that Vimang can significantly inhibit carrageenan- and formalin-induced oedema (in rat, guinea pigs, and mice). Moreover, Garrido et al., 2004 [94], have shown for the first time that Vimang can block TNF*α* (tumor necrosis factor alpha) and inhibit the production of NO (nitric oxide) in macrophages (RAW264.7 and N9) and in mice model of septic shock. Martnez et al., 2000 [95], have evaluated* in vitro* antioxidant effects of Vimang with the help of some commonly accepted assays. Strong radical scavenging activity and a significant inhibition of peroxidation of rat-brain phospholipids by Vimang were observed in this study.

Neuroprotective efficacy of mangiferin in doxorubicin (DOX)-induced rats has been studied by Siswanto et al., 2016 [96]. Brain damage in male Sprague-Dawley rats has been induced by doxorubicin, and mangiferin has been given to brain damage-induced rats for 7 weeks. Results of this study have shown that mangiferin can effectively reverse the brain damage induced by doxorubicin. Cognitive enhancing effects and improvement in memory impairment by* M. indica* fruit pulp extract (ethanol) were studied by Wattanathorn et al., 2014 [97]. To determine cognitive enhancing effects and improvement in memory impairment, male Wistar rats have been administered with the neurotoxin AF64A and given the fruit peel extract. Results of the study have shown increased cholinergic neurons density and decreased oxidative stress in rates, which illustrates possible cognitive enhancing effects of* M. indica* fruit pulp. Neuroprotective activities of methanol and aqueous extracts of* M. indica* leaf have been studied by Kawpoomhae et al., 2010 [98]. Neuroprotective effects of methanol and aqueous extracts were evaluated by determining protection of neuroblastoma cells from H_2_O_2_-induced oxidative damage and results showed that methanol extract and aqueous extract can effectively protect H_2_O_2_-induced neuroblastoma cells from oxidative damage.

Liver, a vital organ in the human body, mainly regulates metabolism and detoxification of toxic substances [166]. It plays an important role by removing reactive oxygen species (ROS) and helps maintains oxidative balance [167]. A number of chemical substances that cause hepatotoxicity by inducing oxidative damage and lipid peroxidation have been identified [168]. Hepatotoxicity is currently treated with drugs that can activate p450 enzyme mechanism either by stopping or inducing the metabolic activity of enzymes [169]. Investigation of phytochemicals with hepatoprotective effects and their mechanism of action has gained much attention. Many authors have investigated hepatoprotective effects of certain plant extracts/pure compounds including* M. indica*. Ebeid et al. 2015 [170] have demonstrated hepatoprotective effects of an aqueous extract of leaves of an* M. indica* variety found in Egypt where they found that the aqueous extract successfully inhibited CCl_4_-induced hepatocellular toxicity in albino rats. Results were further confirmed by analyzing lipid profiles, high-density lipoprotein (HDL), and malondialdehyde (MDA) levels. An* in vitro* study carried out by Hiraganahalli et al. 2012 [171] has shown that methanol/acetone extract of* M. indica* bark can exert hepatoprotective effects in tert-butyl hydroperoxide-induced HepG2 cells in a dose-dependent manner. Hepatoprotective effects of lupeol and aqueous* M. indica* pulp extract (collected from Lucknow, India) have been studied in 7,12-Dimethylbenz[a]anthracene (DMBA)-induced Swiss albino mice. Lupeol and mango* M. indica* extract were found to be effective in the treatment of liver injury caused by oxidative stress [172]. Pourahmad et al. 2010 [173] investigated hepatoprotective effects of an aqueous extract of a mango fruit variety collected from Iran and demonstrated that the extract exerts hepatoprotective effects in cumene hydroperoxide-induced rat hepatocytes. Hepatoprotective effects of an ethanolic extract of kernel of a Thai* M. indica* variety have been also reported by Nithitanakool et al. 2009 [174]. Significant hepatoprotective effects of the ethanolic extract of kernel have been reported in rats with liver injuries induced by carbon tetrachloride (CCl_4_).

### 5.3. Analgesic Effects

Analgesic effects of stem-bark aqueous extract of* M. indica* have been studied by Ojewole, 2005 [175]. Hot-plate and acetic acid test models of pain in mice have been used to study analgesic effects, and results of this study have demonstrated significant analgesic effects in mice with nociceptive pain. Islam et al. 2010 [176], have demonstrated analgesic effects of methanol extract of leaves of* M. indica.* Results have demonstrated a significant reduction in writhing response in an acetic acid-induced writhing response rat model. Garrido et al., 2001 [93], have shown possible analgesic effects of Vimang. Acetic acid-induced abdominal restriction and formalin-induced licking were used to test analgesia. Results of this study have shown that Vimang can exhibit antinociceptive effects in mice. Moreover, a considerable dose-dependent inhibition in formalin-induced pain was also observed in rats after administration of Vimang.

### 5.4. Immunomodulatory Effects of* M. indica*

Immunomodulation is a process that adjusts the immune system of an organism upon any change caused by a foreign agent [177]. Immunomodulation can be of two types, namely immunostimulation and immunosuppression [178]. Immunostimulation includes stimulation of the immune system with immunostimulating agents that activate components of the immune system (macrophages, certain T-lymphocytes and granulocytes) [178]. In immunosuppression, efficiency of the immune system decreases [178]. As clinically used immunomodulating drugs cause serious side effects, it is necessary to discover immunomodulating agents with fewer side effects [179]. Garrido et al. 2005 [180] have shown immunomodulatory activity of bark aqueous extract of a mango variety collected from Cuba. Inhibition of proliferation of T-cells and NF- *κ*B transcription factor were reported in this* in vitro* study suggesting possible immunomodulation. Another study conducted in Cuba with an aqueous extract of mango bark has also shown* in vivo* immunomodulatory effects, where NOS-2, COX-2, IL-1*β*, TNF-*α*, and colony-stimulating factor (GM-CSF) mRNA levels were found to decrease in experimental mice model(s) [181]. Makare et al. 2001 [182] have assessed immunomodulatory effects of an ethanolic extract of mango bark rich in mangiferin in Swiss albino mice. Administration of the ethanolic bark extract increased delayed type hypersensitivity (DTH) and humoral antibody (HA) titer suggesting possible immunostimulation by the extract. Immunostimulatory effects of a mango kernel powder in a species of fish* (Labeo rohita)* infected with* Aeromonas hydrophila* have been studied by Sahu et al. 2007 [183], where increased immunological parameters (lysozyme activity, superoxide anion production, and bactericidal activity) were detected in fish fed with mango kernel powder. Moreover, a recent study has shown that a hexane leaf extract from* M. indica* collected from Varanasi, India, possesses immunomodulatory effects in RAW 264.7 cells. Immunomodulatory effects were confirmed by analyzing intracellular NO levels, where a significant increase in response to the leaf hexane extract was observed. Furthermore, oral administration of the leaf hexane extract has also caused increased white blood cells, hemoglobin concentration, and temporary increase in the size of spleen and thymus in cyclophosphamide-induced myelosuppressed mice, which indicates immunostimulation in bone marrow hematopoietic cells and white blood cells [184].

### 5.5. Antitumoral Effects of* M. indica*

Cancer is considered as one of the major causes of death in the world and any practical solution in fighting this dreadful disease would be very important in public health [185]. It is the main cause of death in economically developed countries and the second leading cause of death in economically developing countries [186]. Cancer is caused by several factors such as chemicals, radiations, tobacco, infectious microorganisms, hormones, gene mutations, and immune conditions [187]. Though, modern surgeries have considerably reduced the cancer death rates, use of radiotherapy, chemotherapy, and hormone therapy treatments cannot completely reduce the number of deaths due to cancer [188]. Plant-based treatments have been used in traditional medicine to treat different diseases including cancer since ancient times and a number of* in vitro* and* in vivo* studies have already been reported in literature to validate these uses [189]. Different organic extracts and decoctions prepared from parts of mango trees and compounds isolated from mango trees have shown anticancer effects.

A recent study by Abbasi et al. 2017 [74] demonstrated antiproliferative effects of fruit peel and pulp of several mango varieties (Royal mango, Thai mango, Egg mango, Luzon Narcissus, Big Tainong, Keitt, Australian mango, and Small Tainong) grown in China. They showed that the acetone extracts of mango peel and pulp exerted antiproliferative effects in HepG2 cells. Another study carried out by Kim et al. 2012 [190] has shown that the ethanolic extract of* M. indica* peel can induce apoptosis in human cervical adenocarcinoma HeLa cells. Apoptotic effects of the peel ethanolic extract have been studied by analyzing expression of apoptosis-related proteins Bax, Bcl-2, Bid, and caspases (3, 8, and 9) in this study. Phytochemical investigation of peel ethanolic extract has revealed that it contains some reported anticancer compounds such as quercetin 3-O-galactoside, gallic acid, linoleic acid, alpha-tocopherol, mangiferin gallate, mangiferin, kaempferol 3-glucoside and quercetin-3-O-arabinopyranoside. Protective effects of ethanolic extracts of mango fruit peel and flesh (a Korean variety) samples in H_2_O_2_-induced cytotoxicity in HepG2 cells have also been studied by this research group [76]. A study carried out by Corrales-Bernal et al. 2014 [191] has shown that aqueous extract of mango fruit flesh possesses antiproliferative effects in human colon adenocarcinoma cell line (SW480) and in mouse model with colorectal cancer. Antiproliferative effects of methanol extracts of peel and flesh of three mango cultivars (Kensington Pride (KP), Nam Doc Mai (NDM), and Irwin (IW)) found in Australia were studied by Taing et al. 2015 [192]. They have demonstrated that peel methanol extract of NDM can only inhibit the proliferation of MCF-7 breast cancer cells. Antitumor effects of mango polyphenols-rich fruit flesh extracts in human breast cancer xenografts mice have been studied by Banerjee et al. 2015 [193]. This study has proven that mango polyphenols rich fruit pulp extract has a potential to target PI3K/AKT pathway in breast cancer. Anticarcinogenic effects of a crude (methanol : acetone : water-1 : 1 : 1) fruit peel extract of some selected mango varieties (Kent, Francis, Atkins, Ataulfo, Tommy, and Haden, found in Brazil) have been evaluated in leukemia (Molt-4), lung (A-549), triple negative breast (MDA-MB-231), prostate (LnCap), and colon (SW-480) cancer cells by Noratto et al. 2010 [194]. Among the studied mango varieties, two (Ataulfo and Haden) were more sensitive to SW-480 and MOLT-4 cells. Moreover, apoptotic effects of Ataulfo and Haden varieties have also been studied in SW-480 cells in this study. Induction of apoptosis by aqueous extract of mango fruit peel rich in lupeol has been carried out in testosterone-induced mouse prostate and human prostate cancer cells (LNCaP) by Prasad et al. 2007 [195]. Antiproliferative effects of two extracts (pectinase and Soxhlet extracts) of mango flesh found in Australia have shown in oestrogen receptor positive (MCF-7) breast cancer cells by Wilkinson et al. 2011 [196].

Induction of oxidative stress mediated apoptosis by ethanolic extract of* M. indica* seed in triple negative breast cancer cells (MDA-MB-231) has been reported by Abdullah et al. 2015 [197]. In this study, apoptotic effects of ethanolic extract of* M. indica* seeds were evaluated by analyzing apoptosis-related marker proteins such as Bax, Bcl-2, cytochrome c, and caspases (3, 8, and 9). Involvement of oxidative stress markers such as reactive oxygen species (ROS), glutathione (GSH), and malondialdehyde (MDA) levels in apoptosis has also been studied. Another study [198] carried out by the same research group reported oxidative stress mediated apoptosis by ethanolic extract of mango seeds in oestrogen receptor positive breast (MCF-7) cancer cells. Nguyen et al. 2016 [199] have demonstrated cytotoxic effects of methanol bark extract of* M. indica* in pancreatic cancer cells (PANC-1). Isolation of two novel cycloartane-type triterpenes, namely, mangiferolate A and mangiferolate B, has also been reported in the same study. Studies on the anticancer effects of mango leaves are limited. Cytotoxic effects of ethanolic leaf extract of an* M. indica* variety grown in Thailand were investigated by Ganogpichayagrai et al. 2017 [200], but a very low cytotoxic potential by ethanolic extract has been reported in all the cancer cell lines tested. Hepatoblastoma (HepG2), gastric carcinoma (Kato-III), bronchogenic carcinoma (Chago K1), ductal carcinoma (BT474), and colon adenocarcinoma (SW 620) cell lines were used in this study. Cytotoxic and apoptotic potential of the bark of two mango varieties (Rata Amba and Kartha Kolomban Amba) grown in Sri Lanka in breast (MCF-7 and MDA-MB-231) and ovarian cancer (SKOV-3) cells has been recently reported by us [201]. In this study we found that methanolic extracts of bark of two mango varieties exert cytotoxic and apoptotic potential in breast and ovarian cancer cells. In addition to these findings, our recent findings have demonstrated that the hexane extract of bark and the chloroform extract of the fruit peel of* Mangifera zeylanica*, a plant endemic to Sri Lanka (Sri Lankan mango), can induce apoptosis in breast and ovarian cancer cells [202, 203]. Results of our studies showed that the hexane extract of bark of* M. zeylanica* has promising cytotoxic effects in breast (MCF-7 and MDA-MB-231) and ovarian cancer (SKOV-3) cells with less cytotoxicity to normal mammary epithelial (MCF-10A) cells [202]. Hexane extract also showed apoptotic effects in these cancer cells. Phytochemical investigation by GC-MS analysis of the active fractions of the hexane extract revealed few unknown compounds and we isolated a new resorcinolic lipid which was cytotoxic to MCF-7 breast cancer cells [50]. Furthermore, we isolated two new halogenated compounds namely chloromangiferamide and bromomangiferic acid from the chloroform extract of* M. zeylanica* bark [52]. Of these chloromangiferamide was cytotoxic only to MDA-MB-231 cells whereas bromomangiferic acid had no cytotoxic activity. Studies with the* M. zeylanica* fruit peel and flesh demonstrated that chloroform extract of fruit peel can induce apoptosis in MCF-7 breast cancer cells through an oxidative stress mechanism. Phytochemical identification of the peel chloroform extract of* M. zeylanica* showed presence of some reported anticancer compounds such as linoleic acid and *α*-tocopherol [203]. Mangiferin is a well-known bioactive xanthonoid found in various parts of the mango tree. A number of studies have been carried out to illustrate antitumoral effects of mangiferin in various cancer cell lines such as breast, lung, ovary, brain, and cervix, and possible antitumoral mechanisms of mangiferin in several cancer cell lines have also been well-documented [204].

### 5.6. Antibacterial Effects of* M. indica*

Resistance to antibiotics has become one of the biggest problems worldwide [205]. Unnecessary use of antibiotics for viral infections, prolong use of antibiotics for diseases, wrong prescriptions given to patients without determining the exact cause of infection, and discontinuation of antibiotics without completing treatments by patients are some of the major causes for occurrence of antibiotic resistance [206]. Approximately half of all deaths in tropical countries are due to bacterial infections [207]. Therefore, discovery of novel antibacterial agents for drug resistant bacteria is essential. A number of studies have proven the antibacterial effects of certain plant crude drugs and natural compounds isolated against drug resistant bacteria [208]. Herbal remedies for bacterial infections have gained much attention recently as they are readily available, cause fewer side effects, and cheap.

Various studies have been conducted with the extracts of roots, leaves, bark, fruit peel and flesh, and kernel of* M. indica* to investigate antibacterial properties. Among these parts, mango kernel and leaves are the most studied parts for antibacterial effects. A study carried out by Mutua et al. 2017 [209] with four* M. indica* varieties grown in Kenya (Apple, Ngowe, Sabine, and Kent) found that the methanol extracts of kernels of Apple and Sabine varieties exert strong inhibitory effects against* Escherichia coli.* Antibacterial effects of hexane, chloroform, benzene, methanol, and water extracts of kernel of an* M. indica* variety found in Tamil Nadu, India, have been reported by Rajan et al. 2011 [210]. The methanolic extract of the kernel was more potent in inhibiting the growth of* Shigella dysenteriae*, a causative agent for diarrhoea. Aqueous kernel extracts of two other* M. indica* varieties (Bagnapalli and Senthura) found in Tamil Nadu (Vellore) were subjected to study antibacterial effects against* Staphylococcus aureus* and* Pseudomonas aeruginosa* by Alok et al. 2013 [211]. Aqueous extracts of Bagnapalli variety exerted a higher effect than the Senthura variety. Antibacterial effects of kernels obtained from three mango varieties (Black Gold, Lemak, and Waterlily) grown in Malaysia were studied by Mirghani et al. 2009 [212]. Kernel samples were extracted to ethanol, methanol, acetone, and phosphate buffer saline and were subjected to antibacterial studies against* S. aureus*,* E. coli*,* P. aeruginosa* and* Bacillus subtilis*. The results of this study showed that the ethanol and methanol extracts of Lemak have the highest antibacterial potential. Inhibitory effects of methanolic kernel extract (rich in polyphenols) of a Japan* M. indica* variety against 43 bacterial species have been evaluated by Kabuki et al. 2000 [213]. Methanolic kernel extract of this variety was found to be more active against Gram-positive bacteria in tested organisms.

A number of studies have also been carried out to evaluate antibacterial effects of different extracts of* M. indica* leaves worldwide. A recent study carried out by Diso et al. 2017 [214] showed that chloroform and aqueous extracts of leaves of an* M. indica* variety collected in Nigeria possess antibacterial effects against isolates of* S. aureus.* Chloroform extract of leaves was found to be more inhibitive than the aqueous extracts tested.* In vitro* antibacterial effects of water, methanol, and acetone extracts of leaves of another* M. indica* variety grown in Nigeria have been tested on* Shigella flexneri*,* E. coli*,* S. aureus*,* Streptococcus pyogenes*,* Bacillus cereus*,* P. aeruginosa*,* Streptococcus pneumoniae*,* Proteus mirabilis*, and* Salmonella typhi* by Doughari and Manzara, 2008 [215]. Islam et al. 2010 [176] showed the antibacterial effects of leaf ethanol extract of a mango variety found in Bangladesh. Growth inhibition of Staphylococcus* aureus*,* Streptococcus agalactiae*,* B. cereus*,* Bacillus megaterium*,* B. subtilis*, and* Lactobacillus bulgaricus* bacterial species by methanolic leaf extract was observed in the study. An acetone extract of the leaves of an* M. indica* variety grown in Pakistan exerted a strong inhibition of growth of multidrug resistant* S. typhi* [216]. Antibacterial effects of several mango leaf extracts grown in India have been proven in several bacterial species by Bharti, 2013 (*M. indica* variety collected from Rewa district, Madhya Pradesh) [217], Chandrashekar et al. 2014 (mango variety collected from Bhopal, Madhya Pradesh) [218], Madduluri et al., 2013 (collected from Andhra Pradesh) [219], and Sharwat et al., 2013 (collected from Meerut region) [220]. Bhati, 2013 found that hexane and hexane/ethyl acetate extracts of leaves exert promising antibacterial effects against* Mycobacterium tuberculosis*,* Enterobacter aerogenes*,* and S. pyogenes*. Chandrashekar et al. 2014 reported that ethanolic extract of leaves can inhibit the growth of* Streptococcus mutans.* Methanolic and ethanolic extracts of leaves of* M. indica* were subjected to antibacterial studies against* E. coli*,* klebsiella pneumoniae*,* Salmonella typhimurium*,* S. aureus,* and* B. cereus* in the study conducted by Madduluri et al. 2013. They observed strong inhibitory effects by methanolic and ethanolic extracts against* B. cereus*. Sharwat et al. 2013 found that methanol, ethanol, and benzene extracts of* M. indica* leaves can effectively inhibit the growth of* Pseudomonas fluorescens.* Furthermore, antibacterial effects of ethanolic and methanolic extracts of mango seeds have been subjected to antibacterial effects by Awad El-Gied et al. 2012 [221]. 25 different bacterial strains have been used in this study and out of the 25 strains,* Mycobacterium smegmatis* showed highest inhibition after exposure to ethanolic and methanolic extracts. Antibacterial effects of mango sap have been determined by Negi et al. 2002 [222]. Aqueous and nonaqueous phases of mango sap obtained from four* M. indica* varieties (Mallika, Totapuri, Raspuri, and Seedling) have been evaluated for antibacterial activity against* S. aureus*,* E. coli*,* B. cereus,* and* P. aeruginosa* and the nonaqueous phase of all four* M. indica* varieties was found to be more inhibitory against* B. cereus*, whereas aqueous extracts showed no inhibitory effects. A study carried out with pet ether, ethyl acetate, ethanol, and water extracts of roots of the an* M. indica* variety collected form Karnataka, India, by Latha et al. 2011 [223] has proven antibacterial effects. Ethanol extract of roots of studied* M. indica* variety was found to be more inhibitory against* B. subtilis*,* E. coli*, and* K. pneumoniae.* Silver nanoparticles (loaded onto nonwoven fabrics) prepared from aqueous extract of mango peel have been subjected to antibacterial effects by Yang and Li, 2013 [224]. The results of this study revealed that prepared nanoparticles can effectively inhibit the growth of* E. coli*,* S. aureus*, and* B. subtilis*. A decoction prepared from ripe and unripe mango peel and seeds has been used to study antibacterial effects by Rakholiya et al. 2013 [225]. Among twenty bacterial species used in this study,* Micrococcus flavus* was found to be more susceptible to the decoctions prepared from ripe seeds, ripe peel, and unripe seeds.* In vitro* antibacterial effects of four extracts (aqueous, ethanol, methanol, and acetone) prepared from mango flowers have been studied by Verma et al. 2015 [226] against six pathogenic bacterial strains and methanolic and ethanolic extracts of flowers have shown highest inhibition against* S. typhi.* Singh et al. 2015 [51] have shown antibacterial effects of bark of a mango variety found in India. Hexane and methanol extracts obtained from Soxhlet extraction of bark have exerted promising antibacterial effects against* B. subtilis*,* S. typhi*,* P. aeruginosa*,* E. coli*,* and S. aureus.* Results of antibacterial assays have shown that hexane extract was more active against tested bacterial species. Antibacterial potential of aqueous extract of mango bark has also been carried out by Chidozie et al. 2014 [227].* E. coli*,* P. aeruginosa*,* Proteus vulgaris*,* Streptococcus faecalis*,* S. typhi*, and* Shigella* have been included in this and results of this study have illustrated that aqueous extract of mango bark was inhibitory to all bacterial species tested except* S. faecalis*.

### 5.7. Antifungal Effects of* M. indica*

Fungal diseases have been identified as an important health problem nowadays [228].* Candida*,* Aspergillus,* and* Cryptococcus* species are known to causes many fungal diseases worldwide [228].* Candida albicans* is reported to be the most common pathogen in fungal infections [229]. Although fungal infections are common, few antifungal drugs are currently used to treat infections [230]. Therefore, the identification of novel drugs as antifungal agents is necessary. Plant-based treatments and natural compounds derived from plants have been identified as ideal drug leads for fungal diseases. A number of pharmacological investigations have confirmed antifungal effects of organic/aqueous extracts of different parts of* M. indica*. A study was conducted by Muazu et al. 2017 [231] to find antifungal effects of ethanol extract of leaves of an* M. indica* variety collected in Nigeria. Both extracts tested have shown moderate antifungal effects against* Fusarium oxysporum*,* Fusarium avenaceum*, and* Pythium aphanidermatum*. Moreover, Islam et al. 2010 [176] have evaluated antifungal effects of an ethanolic extract of the leaves of* M. indica* against three fungal species namely* Aspergillus ochraceus*,* Aspergillus niger*, and* Aspergillus ustus*. Moderate antifungal activity against studied three fungal species has been reported in this study. Antifungal effects of an aqueous extract of leaves of an* M. indica* variety found in Mexico have been evaluated by Bautista Banos et al. 2002 [232] and results showed a moderate inhibition of fungal strain* Colletotrichum gloeosporioides* by the aqueous extract. A recent study conducted with seed extracts of four* M. indica* varieties (Keitt, Sensation, Gomera-3, and peel) found in Spain showed that the extracts inhibited growth of all 18-fungal species tested [233]. Among these species* Candida parapsilosis*,* Candida glabrata*, and* Lodderomyces elongisporus* have exhibited a higher sensitivity towards tested extracts. However, authors have not included the extraction method and type of extracts used to determine antifungal effects. Mango kernel has also been reported to possess antifungal effects. A study carried out by Mutua et al. 2017 [209] has reported methanolic kernel extracts of four mango varieties grown in Kenya (Apple, Ngowe, Sabine, and Kent) exert inhibitory effects against* C. albicans*. Extracts of Apple, Ngowe, and Sabine showed more inhibitory effects than Kent against* C. albicans.* Antifungal effects of* M. indica* bark have also been included in previously mentioned studies [231, 232]. Moderate antifungal activity has been reported for ethanol and methanol extracts [231] and aqueous extract [232] of* M. indica* bark.

### 5.8. Anthelmintic Effects of* M. indica*

Helminth infections which are caused by parasitic worms are commonly seen in tropical regions [234]. They live either as parasites or in some cases in a free-living form [234]. Intestinal nematodes (IN) or soil-transmitted helminths (STH) are the most common types of nematodes [235]. It has been estimated that approximately 30% of world population is primarily infected with helminth parasites annually [236]. Development of resistance to helminth parasites has become a major problem in the treatment of the helminth infections [236]. Hence, discovery of natural remedies that can target helminth infections is important. Anthelmintic effects of extracts of different parts of* M. indica* have been studies in several* in vitro* studies. Anthelmintic effects of petroleum ether, ethyl acetate, and ethanol extracts of roots of two* M. indica* varieties (*M. indica* L. Var. Thotapuri and* M. indica* L. Var. Neelam) collected from Karnataka, India, were investigated by Latha et al. 2012 [237]. They showed dose-dependent anthelmintic effects of all three extracts of the two mango varieties used against earthworm* Pheretima posthuma* with a higher activity for* M. indica* L. Var. Thotapuri than for* M. indica* L. Var. Neelam. Sujon et al. 2008 [238] have evaluated anthelmintic effects of an ethanolic extract of roots of an* M. indica* variety collected from Bangladesh. Moderate anthelmintic effects were seen against adult nematodes collected from the gastrointestinal tract of goats. Anthelmintic effects of aqueous extract of mango fruit against intestinal nematode* Strongyloides stercoralis* have been studied by El-Sherbini and Osman, 2013 [239]. Aqueous extract of* M. indica* fruits have shown 100% inhibition of* S. stercoralis* larval development. A study carried out by García et al. 2003 [240] demonstrated anthelmintic effects of an aqueous extract of bark of* M. indica* in* Trichinella spiralis* where significant reduction in parasite larvae and a reduction of serum specific antitrichinellaIgE were reported.

### 5.9. Antiplasmodial Effects of* M. indica*

Complete eradication of malaria appears to be a major challenge in the world due to the development of resistance to antimalarial drugs [241]. Many parasites in genus* Plasmodium* cause malaria and* Plasmodium falciparum* is the most predominant in genus* Plasmodium* [242]. It has been estimated that malaria affects approximately 280–290 million people annually [242]. Quinine and artemisinin are naturally derived antimalarial drugs, which have been used for almost 400 years in the treatment of malaria [243]. However,* P. falciparum* has developed complete resistance to almost all the antimalarial drugs in clinical use [243]. Therefore, it is of paramount importance to investigate novel treatment methods/drugs which can target the malaria parasites. Despite latest inventions of new drugs by pharmaceutical companies, medicinal plants have gained much interest as novel sources for antimalarial drugs. Studies with antiplasmodial effects of mango are limited in literature. A study carried out by Awe et al. 1998 [244] has shown antiplasmodial effects of a bark methanol extract of a mango variety collected from Nigeria. The methanol extract has exhibited significant antiplasmodial effects against malaria parasite,* Plasmodium yoelii nigeriensis*. Zirihi et al. 2005 [245] have also shown some mild inhibitory effects of an ethanolic extract of mango bark collected from Ivory Coast on* P. falciparum*. Bidla et al. 2004 [246], on the other hand, have shown that methanol/chloroform extract of leaves of a mango variety collected from India possesses moderate antiplasmodial effects in* P. falciparum.*

### 5.10. Antihyperlipemic Effects of* M. indica*

Hyperlipidemia is considered as a major reason for atherosclerosis and coronary heart disease [247]. Coronary heart disease is the main cause of death in the world [247]. Scientific investigation of herbal remedies for antihyperlipemic effects will give a strong support for the development of drugs for hyperlipidemia. A recent study by Gururaja et al., 2017 [248], reported cholesterol lowering effects of a methanolic extract of* M. indica* leaves in albino Wistar rats. A significant decrease in plasma cholesterol levels has been observed in rats administrated with cholesterol in this study. Results of the studies conducted with aqueous extracts [249, 250] and ethanolic extracts [251, 252] of* M. indica* leaves have shown promising antihyperlipemic effects in hyperlipemic rat models. Another study conducted by Vasant and Narasimhacharya, 2011 [253], has reported that feeding of mango fruit powder to hyperlipemic rats can significantly reduce serum cholesterol levels, very low-density lipoproteins (VLDL), and triglycerides (TG). A study conducted by Dineshkumar et al. 2010 [254] with 828 type 2 diabetes patients with high serum cholesterol levels, living in Gopali, India, has shown that consumption of aqueous extract of mango bark can significantly reduce serum total cholesterol level.

### 5.11. Antidiabetic Effects of* M. indica*

Diabetes mellitus is a metabolic disease resulting from a defect in insulin action or secretion [255]. It has now become a major health problem affecting 442 million people worldwide [256]. 90% of diabetes cases are type 2 and the remainder is type 1 [257]. Blood glucose homeostasis is the key to prevent diabetes associated complications such as cardiovascular diseases, kidney diseases, eye problems, and peripheral neuropathy [258]. Several plant-based remedies have been used to treat type 2 diabetes in traditional medicine and a number of pharmacological studies have been conducted to validate these claims [259, 260]. Fruit peel, flesh, seed kernel, leaves, and bark of* M. indica* have been extensively studied for their antidiabetic properties. Gondi and Prasada Rao, 2015 [261] have showed that an ethanolic extract of mango fruit peel can successfully reduce blood glucose level in streptozotocin-induced diabetic rats. Significant decrease in fructosamine and glycated hemoglobin, which are considered as status indicators of diabetes, has also been observed after treatment with the ethanolic extract of mango peel. Another study carried out by Gondi et al. 2015 [262] with* M. indica* fruit peel powder showed a significant reduction of blood glucose level and diabetes associated complications in rats. Similar results have been obtained in a study carried with a flour prepared from mango fruit pulp [263]. Irondi et al., 2016 [264], showed that flour supplement prepared with mango kernel effectively reduced blood glucose level in diabetes rats. Improvement in liver function, blood glucose level, hepatic glycogen, lipid profile, and hepatic and pancreatic malonaldehyde was observed in diabetic rats supplied with flour supplement.

Several studies on antidiabetic effects of* M. indica* leaves have been conducted. Antidiabetic efficacy of methanolic leaf extracts of young and matured leaves of* M. indica* has been evaluated by Mohammed and Rizvi, 2017 [265]. In this study authors found that young leaves were more effective than matured leaves as antidiabetic. Evaluation of antidiabetic effects, by determining inhibition rates of yeast, rat alpha-glucosidase, and porcine pancreatic alpha-amylase, has shown that ethanolic leaf extracts (Thai and Indian mango) have antidiabetic effects [200, 266]. Aderibigbe et al. 1999 [267] found that aqueous extract of* M. indica* leaves can significantly reduce blood glucose level in streptozotocin-induced diabetic rats. Tanko et al. 2012 [268], Mangola, 1990 [269], Miura et al., 2001 [270], and Waheed et al., 2006 [271], have also proven hypoglycemic effect of aqueous extract of* M. indica* leaves in diabetic rats. Sharma et al., 1997 [272], have demonstrated hypoglycemic potential of ethanolic extract of mango leaves in normal and streptozotocin-induced diabetic rats. They have successfully shown significant antihyperglycaemic effects in diabetic rats when supplied with ethanolic extract of* M. indica* leaves. A study conducted by Wadood et al. 2000 [273] found antidiabetic effects of alcoholic extract of the leaves of* M. indica* in rabbits. Studies carried out with water extracts of* M. indica* stem-bark by Bhowmik et al., 2009 [274], and Ojewole, 2005 [175], have also proven antihyperglycaemic effects in type 2 diabetic rat model.

### 5.12. Gastroprotective Effects of* M. indica*

Peptic ulcers are mainly present in the lining of stomach or in the duodenum [275]. Nonsteroidal anti-inflammatory drugs, mental or physical stress, alcohol, diet, life style, and antibiotics are considered as main causes of peptic ulcers [276]. Peptic ulcers are usually treated with proton pump inhibitors that reduce the secretion of gastric HCl [276]. As peptic ulcer cases are rising at an alarming rate worldwide, it is necessary to discover novel methods or drugs that can effectively reduce peptic ulcers. Assessment of gastroprotective effects of different extracts of* M. indica* has been carried out. A study conducted by Lima et al. 2006 [277] found that a decoction prepared from* M. indica* flowers can significantly increase gastroprotective properties in an experimental rat model by reducing gastric juice volume and acidity. Furthermore, Severi et al. 2009 [278] have shown that a decoction prepared from leaves of* M. indica* reduces gastric lesions induced by HCl, ethanol, and nonsteroidal anti-inflammatory drugs in experimental rat models. Antiulcer potential of ethanol and petroleum ether extracts prepared from mango leaves has also been reported by Neelima et al. 2012 [279]. Akindele et al. 2012 [280] who assessed gastroprotective effects of a drug formulation (DAS-77) which comprises* M. indica* bark and papaya roots showed a significant reduction of gastric ulcers after feeding with DAS-77 in rat models. Antiulcer activity of an ethanolic extract of mango kernels and in combination with vitamin C, ZnSO_4,_ and menadione in pylorus ligation and ethanol-induced ulcers in rat models was evaluated by Nethravathi K et al. 2015 [281]. Considerable reduction in gastric volume, ulcer score and index, and total acid output was observed after administration of ethanolic extract and the above drug combinations.

There is a good agreement that exists between pharmacological properties of crude extracts of different parts of* M. indica* and biological effects of pure compounds isolated from* M. indica*. Common compounds found in various parts (bark, leaves, and fruits) of* M. indica* such as mangiferin, derivatives of mangiferin, gallic acid, catechin, quercetin, *β*-carotene, shikimic acid, and kaempferol have been reported to possess antioxidant effects in several* in vitro* and* in vivo* studies [54–58, 61, 62]. Studies that have shown antioxidant effects of crude extracts of* M. indica*, [73–83], further suggest a striking correlation for the presence of these antioxidant compounds in those tested extracts. Catechin, mangiferin, gallic acid, epigallocatechin gallate, friedelin, humulene, kaempferol, and quercetin are well-known reported anticancer compounds present in mango [54–58, 61, 62]. Available studies that show anticancer effects of different crude extracts (leaves, bark, and fruits), [190–202], strongly correlate the presence of these compounds in crude extracts of* M. indica*. Alkylresorcinols, epigallocatechin gallate, mangiferin, friedelin, gallic acid, and quercetin have been reported to possess anti-inflammatory effects in various* in vitro* and* in vivo* studies [54–58, 61, 62]. Occurrence of these anti-inflammatory compounds in* M. indica* has been well-documented and reported pharmacological studies which illustrate anti-inflammatory effects [88–98, 166–174] of* M. indica*'s crude extracts strongly correlate pharmacological effects and chemical composition. Apart from the abovementioned compounds, a large number of chemical compounds have been isolated and reported from different parts (fruits, bark, leaves, flowers, and roots) of* M. indica*. Some major compounds isolated or identified by gas chromatography–mass spectrometry (GC-MS) and liquid chromatography–mass spectrometry (LC-MS) from different parts of mango tree and reported biological effects of those pure compounds have been listed in Table 2. According to the information present in Table 2, it is clear that common mango compounds such as mangiferin, quercetin, catechin, and kaempferol possess a wide range of pharmacological properties.

## Figures and Tables

**Table 1:** Ethnomedicinal use of different parts of *M. indica *in the world.

Country	Part(s) used	Ethnomedicinal use	Reference(s)
Bangladesh	Bark	Diarrhoea, gastric disorders, asthma, mouth sores, liver diseases, urinary tract infections, diabetes, rheumatism, leucorrhea, bleeding hemorrhoids, lung hemorrhage, nerve disorders, syphilis, cough, and jaundice. Resins of the mango bark have been used for the treatment of cracked skin and feet	[16]
	Seeds, fruit, and kernel	Urethrorrhea, vaginopathy, dysentery, diarrhoea, ophthalmia, and hemorrhage in lungs, uterus, and intestine	
	Roots	Ulcers, syphilis, and leucorrhea.	
	Flowers	Ulcers, diarrhoea, hemorrhage, anemia, dyspepsia, and dysentery.	
	Leaves	Hemorrhages, diarrhoea, ulcers, dysentery, cough, gall bladder and kidney diseases, wounds, throat diseases, and hiccups. The ash of burnt mango leaves has been used as a local application on burns and scalds. According to available reports, leaves have commonly been used for diabetes in the form of a decoction or powder	

Benin	Bark	Hypotension and anemia	[17]

Brazil	Bark	Scabies/itch	[17]

Canary Islands	Bark	Diarrhea	[17]

Cuba	Bark	Mouth sores, tooth pain, cancer, diabetes, asthma, gastric disorders, and lupus	[17]

Fiji	Bark	Syphilis	[17]

Ghana	Bark	Hypertension and diabetes	[18]

Guyana		Gastric disorders and diarrhoea	[17]

Haiti	Bark	Hepatic disorders	[17]

India	Bark	Diabetes, gastric disorders, asthma, mouth sores, leucorrhea, bleeding hemorrhoids, lung hemorrhage, nerve disorders, syphilis, cough, and jaundice	[9, 19–23]
	Seeds, fruit, and kernel	Ophthalmia, hemorrhage in lungs, uterus and intestine, urethrorrhea, vaginopathy, dysentery, and diarrhoea	
	Roots	Ulcers, syphilis, and leucorrhea	
	Flowers	Ulcers, diarrhoea, hemorrhage, anemia, dyspepsia, and dysentery	
	Leaves	Diarrhoea, ulcers, diabetes, dysentery, cough, gall bladder and kidney diseases, hemorrhages, wounds, diseases in throat and hiccups, burns, and scalds	

Madagascar	Bark	Liver obstruction	[17]

Mali	Bark	For vomiting	[17]

Nicaragua	Bark	Wounds	[17]

Nigeria	Leaves	Leaf decoctions have been commonly used to treat diabetes and malaria	[24, 25]

Pakistan	Bark	Asthma, bronchitis, cough, and throat problems	[26, 27]
	Leaves and seeds	Earache and vomiting	

Peru	Leaves	Bronchitis, colds, and inflammation	[28]

Senegal		Mouth sores, toothache, dysentery, and diarrhoea	[17]

Sri Lanka	Bark	Menorrhagia, leucorrhea, piles, and hemorrhages of the lungs and intestine	[29]
	Leaves	Diseases of the lungs, coughs, and asthma	
	Flowers	Diarrhoea, dysentery, and gleet	

Tanzania	Bark	Toothache	[17]

Tonga	Bark	Dysmenorrhoea	[17]

**Table 2:** Some common phytochemicals isolated from *M. indica* and their reported biological activities.

Reported pharmacological effects of pure compounds/ crude extracts of *M. indica*	Compounds responsible for reported pharmacological activity	Part(s) used to isolate
Cytotoxic and apoptotic effects [53–56, 99–116]	29-Hydroxy mangiferonic acid	Bark [117], resin [59]
	3,4-dihydroxybenzoic acid (protocatechuic acid)	Bark [118], fruit peel [30]
	Catechin	Bark [118] and leaves [119]
	Elemene	Flower [60], leaves [60], and bark [120]
	Epigallocatechin gallate	Leaves [119], bark [121]
	Ethyl gallate	Flower [60]
	Friedelin	Bark [59, 60]
	Gallic acid	Seed [122], bark [118]
	Humulene	Leaf and flower [60]
	Kaempferol	Fruit [56–58]
	Linalool	Flowers [60], leaves [60], and fruits [123]
	Mangiferin	Bark, leaves, and fruit [17, 54, 55]
	Methyl gallate	Flower [60]
	Mono(2-ethylhexyl) ester	Bark [51]
	N-octyl gallate	Flower [60]
	N-propyl gallate	Flower [60]
	Quercetin	Bark [113–115], fruit [113–115], and leaves [113–115]
	*β*-carotene	Fruit [61]

Anti-inflammatory effects [53–56, 112, 124–128]	5-(11′Z-Heptadecenyl)-resorcinol and 5-(8′Z, 11′Z-heptadecadienyl)-resorcinol	Fruit [124]
	Epigallocatechin gallate	Leaves [119], bark [121]
	Friedelin	Bark [59, 60]
	Gallic acid	Seed [122], bark [118]
	Humulene	Leaf and flower [60]
	Kaempferol	Fruit [56–58]
	Mangiferin	Bark, leaves, fruit [17, 54, 55]
	Shikimic acid	Bark [61], fruit [129]

*Antioxidant effects* [53–56, 104, 106, 130–134]	3,4-Dihydroxy benzoic acid (protocatechuic acid)	Bark [118], fruit peel [30]
	Catechin	Bark [118] and leaves [119]
	Ethyl gallate	Flower [60]
	Gallic acid	Seed [122] bark [118]
	Kaempferol	Fruit [56–58]
	Linalool	Flowers [60], leaves [60], fruits [123]
	Mangiferin	Bark, leaves, fruit [17, 54, 55]
	Methyl gallate	Flower [60]
	N-octyl gallate	Flower [60]
	N-propyl gallate	Flower [60]
	Quercetin	Bark [113–115], fruit [113–115] and leaves [113–115]
	Shikimic acid	Bark [61], fruit [135]
	*β*-carotene	Fruit [61]

*Antibacterial effects* [51, 53–56, 106, 113–115, 118, 136–142]	3,4-Dihydroxy benzoic acid (Protocatechuic acid)	Bark [118], fruit peel [30]
	3-Chloro-N-(2-phenylethyl) propanamide	Bark [51]
	9,12-Tetradecadiene-1-ol-acetate	Bark [51]
	Benzoic acid	Bark [118]
	Catechin	Bark [118] and leaves [119]
	Gallic acid	Seed [122] bark [118]
	Kaempferol	Fruit [56–58]
	Linalool	Flowers [60], leaves [60], fruits [139]
	Mangiferin	Bark, leaves, fruit [17, 54, 55]
	Methyl gallate	Flower [60]
	N-Heneicosane	Bark [60]
	N-Propyl gallate	Flower [60]
	Quercetin	Bark [113–115], fruit [113–115] and leaves [113–115]

*Antifungal effects* [113–115, 143–147]	Benzoic acid	Bark [118]
	Catechin	Bark [118] and leaves [119]
	Mangiferin	Bark, leaves, fruit [17, 54, 55]
	Nerol	Leaf and flower [60]
	N-Pentyl gallate	Flower [60]
	N-Propyl gallate	Flower [60]
	Quercetin	Bark [113–115], fruit [113–115] and leaves [113–115]

*Antiviral effects* [54, 55, 148–151]	Catechin	Bark [118] and leaves [119]
	Isomangiferin	Bark [60] and leaves [152]
	Mangiferin	Bark, leaves, fruit [17, 54, 55]
	Methyl gallate	Flower [60]
	N-Pentyl gallate	Flower [60]

*Antidiabetic effects* [54–58, 106, 153]	3,4-Dihydroxy benzoic acid (Protocatechuic acid)	Bark [118], fruit peel [30]
	Gallic acid	Seed [122] bark [118]
	Kaempferol	Fruit [56–58]
	Mangiferin	Bark, leaves, fruit [17, 54, 55]

*Antimalarial activity* [51, 54, 55]	3-Chloro-N-(2-phenylethyl) propanamide	Bark [51]
	Mangiferin	Bark, leaves, fruit [17, 54, 55]

*Antiobesity activities* [53, 106, 113–116, 154–156]	Epigallocatechin gallate	Leaves [119], bark [121]
	Friedelin	Bark [59, 60]
	Gallic acid	Seed [122] bark [118]
	Mono(2-ethylhexyl) ester	Bark [51]
	Quercetin	Bark [113–115], fruit [113–115] and leaves [113–115]
	*β*-carotene	Fruit [61]

*Immunomodulatory* [54, 55, 116]	Mangiferin	Bark, leaves, fruit [17, 54, 55]
	*β*-carotene	Fruit [61]

*Neuroprotective/analgesic effects/aphrodisiac effects/analgesic* [54, 55, 113–115, 126, 157–159]	Friedelin	Bark [59, 60]
	Linalool	Flowers [60], leaves [60], fruits [139]
	Mangiferin	Bark, leaves, fruit [17, 54, 55]
	Nerol	Leaf and flower [60]
	Quercetin	Bark [113–115], fruit [113–115] and leaves [113–115]
	Ocimene	Flower and leaves [60, 61]

*Effects on PC12 tyrosine kinase activity* [160]	Catechin	Bark [118] and leaves [119]

*Antifibrotic effects* [161]	Elemene	Flower [60], leaves [60] and bark [120]

*Hemolytic activity* [104]	Ethyl gallate	Flower [60]

*Antipyretic activity* [126]	Friedelin	Bark [59, 60]

*Anthelminthic effects* [54, 55]	Mangiferin	Bark, leaves, fruit [17, 54, 55]

*Cardioprotective* [54, 55]	Mangiferin	Bark, leaves, fruit [17, 54, 55]

*Antiamoebic* [54, 55]	Mangiferin	Bark, leaves, fruit [17, 54, 55]

*Antiallergic* [54, 55]	Mangiferin	Bark, leaves, fruit [17, 54, 55]

*Bronchodilatory effects* [54, 55]	Mangiferin	Bark, leaves, fruit [17, 54, 55]

*Lipolytic effects* [54, 55]	Mangiferin	Bark, leaves, fruit [17, 54, 55]

*Effects on cytoplasmic maturation of oocytes* [162]	Mono(2-ethylhexyl) ester	Bark [51]

*Inhibitive effects on hyaluronidase and collagenase* [163]	N-Octyl gallate	Flower [60]

*Nematicidal activity* [164]	Ocimene	Flower and leaves [60, 61]

*Effects on blood pressure* [53, 113–115]	Quercetin	Bark [113–115], fruit [113–115] and leaves [113–115]

*Anticoagulant/antithrombotic* [165]	Shikimic acid	Bark [61], fruit [129]
